# The King’s Lace Bug *Recaredus rex* Distant, 1909 (Hemiptera: Heteroptera: Tingidae): Systematic Position, First Palaearctic and Afrotropical Records, and Ecological Niche Modelling

**DOI:** 10.3390/insects13060558

**Published:** 2022-06-19

**Authors:** Barbara Lis, Anna Zielińska, Jerzy A. Lis

**Affiliations:** Institute of Biology, University of Opole, Oleska 22, 45-052 Opole, Poland; canta@uni.opole.pl (B.L.); anna.zielinska2@uni.opole.pl (A.Z.)

**Keywords:** lace bugs, systematic position, new tribal assignment, distribution, niche modelling, feeding habit, Ghana, India, Iran, Palaeotropics

## Abstract

**Simple Summary:**

Lace bugs (Tingidae) are known for their spectacular bodily appearance; the pronotum and hemelytra of the adult specimens are lacelike, with a delicate network of areolae that resemble lace. The species are phytophagous and always feed on a specific plant or groups of closely related plants. Therefore, they are recognised as mono- or oligophagous bugs, whose feeding activities may cause significant injury to plant pods and leaves. Lace bugs are distributed worldwide and reported in all continents except Antarctica. Although most of the lace bug species are distributed in a particular zoogeographical region, some genera are widely known from the Holarctic region. However, species with a Palaeotropical distribution are scarce. In this study, based on new records and ecological niche modelling, we indicate that *Recaredus rex*, one of the most enigmatic lace bugs, has a possible Palaeotropical distribution. Moreover, we hypothesise that *R*. *rex* is an oligo- or polyphagous species. In addition, the systematic position of the genus *Recaredus* is discussed.

**Abstract:**

The systematic position and actual distribution of *Recaredus rex*, for a long time one of the most enigmatic lace bug genus and species, is very obscure because only the type specimen and three other individuals from India are known to date. In the present paper, we report the first records of *R. rex* from the Palaearctic region (Iran) and tropical Africa (Ghana). Based on the occurrence localities and climatic variables, we predict potentially useful ecological niches for this species using Maxent software. The areas with the best environmental conditions for *R. rex* indicated in our studies suggest its possible Palaeotropical distribution. Moreover, we regard these results as a good starting point for further searches for specimens of this species. This might help verify the hypothesis of the broad Palaeotropical distribution of *R. rex* and its oligo- or polyphagy. In addition, the lace bug genus *Recaredus*, based on the diagnostic characteristics provided for the tribe Acalyptaini, and the structure of *aedeagus,* is transferred from the tribe Ypsotingini to the Acalyptaini. A key to all genera currently included in the latter tribe is also provided.

## 1. Introduction

*Recaredus rex* was described by Distant [1] from West Bengal (India) as a genus and species new to science and was named after the Visigothic King of Hispania and Septimania (Reccared I or Recared I (559–601); in Latin: *Reccaredus* or *Recaredus*, *rex* Visigothorum) [2,3,4].

This Oriental genus was diagnosed, besides other characteristics, as having a “Head distinctly produced and deflected between the bases of the antennae, and also with a distinct lateral curved robust spine between the antennae and eyes” [1,5]. This unique head armoury (bringing to mind a crown worn by the King Recared I in his portrait [6]) most probably inspired Distant to give such an unusual name to this new lace bug.

For a long time, the species was known only from its original short description [1] repeated in the “Fauna of British India” [5], supplemented by a line drawing of the type specimen. In its description, Distant [1,5] suggested that “this genus apart from the structure of the head has a considerable resemblance to Acalypta Westwood, 1840, a Palaearctic genus”. No type material or other specimens of this species have been recorded since its original description. Therefore, *R. rex* was regarded as one of the most enigmatic Oriental lace bugs [7].

However, all authors [8,9,10,11] hypothesised the systematic position of the genus within the family Tingidae based only on its original description and without any explanation for such acts. In the first checklist of the Tingidae genera of the world [8], *Recaredus* was placed within the subfamily Tinginae. Three years later, Drake [9] transferred this genus from the Tinginae to the subfamily Cantacaderinae, where it was maintained during the revision of the lace bug genera of the world [10]. However, a few years later [11], *Recaredus* was reassigned to the subfamily Tinginae and classified within the tribe Tingini, but without any comments or explanations.

Only in the 21st century was the type material of *R*. *rex* discovered [7]. Moreover, three other specimens from India were studied, which enabled redescribing the genus and species and disclosing their systematic position within the family [7]. As a result of those studies, *R*. *rex* was suggested to represent the tribe Ypsotingini (*sensu* Froeschner [12]), being closely related to *Derephysia* Spinola, 1837 and *Kalama* Puton, 1876 (both representing the tribe Ypsotingini at that time). However, Golub and Golub [13] indicated that three genera of Ypsotingini (*Derephysia*, *Kalama*, *Dictyonota* Curtis, 1827) and the genus *Acalypta* Westwood, 1840 (classified to date within the tribe Tingini) have several essential features in common. Therefore, they united those four closely related genera into a tribe under the resurrected valid name Acalyptini [Acalyptaini; ICZN Case 3813] [14].

Since we were able to study the samples of *R*. *rex* from India and recently discovered its Afrotropical and Palaearctic specimens, we decided to verify whether the genus *Recaredus* should also be classified within the tribe Acalyptaini. Moreover, its new country records suggest a not strictly Oriental but much broader area of occurrence. Because *R*. *rex* specimens were found in the territory of three countries that are not directly adjacent, we considered generating the species potential distribution map using the ecological niche modelling (ENM) methods. Ecological niche modelling (ENM) is a method that allows an area of high habitat suitability to be estimated for a species based on data on its occurrence and environmental conditions [15,16].

In true bugs (Hemiptera: Heteroptera), ENM has also been used, but not so often. This method was applied several times to invasive alien species [17,18,19,20,21] and agricultural pests [22,23] and incorporated in ecological studies of various types [24,25,26,27,28,29,30,31].

## 2. Materials and Methods

### 2.1. Specimens Examined

**Figure 1:**
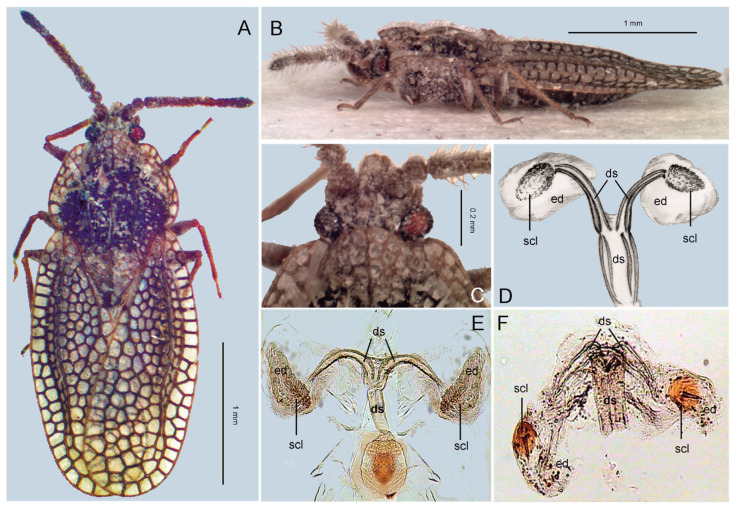
(**A**–**C**) One of two specimens of *Recaredus rex* sampled during the Czechoslovak–Iranian entomological expedition to Iran in 1977. (**A**) Habitus, dorsal view. (**B**) Habitus, lateral view. (**C**) Head, dorsal view. (**D**–**F**). *Ductus seminis* (ds), *endosomal diverticula* (ed) and endosomal sclerites (scl). (**D**) *Recaredus rex* (after [7], modified). (**E**) *Derephysia foliacea* (Fallén, 1807). (**F**) *Kalama tricornis* (Schrank, 1801) (after [33], modified).

Indian specimens of *R*. *rex* come from the entomological collection at the National Pusa Collection (New Delhi, India—NPCI). The African specimen from Ghana belongs to the Museum and Institute of Zoology, Polish Academy of Sciences (Warsaw, Poland—ZMPA). The Iranian specimens (Figure 1A–C) were found in the entomological collection of the National Museum of Natural History, Department of Entomology (Prague, Czech Republic—NMPC), and were sampled during the Czechoslovak–Iranian entomological expedition to Iran [32].

### 2.2. Photograph Preparation

All images of the studied specimens were captured with a Moticam 1000 digital camera mounted to an Olympus SZX10 microscope using an Images Plus 2.0 software (Motic Asia, Hong Kong, China). Multiple focal planes were merged using Helicon Focus 7.7.5 software (Helicon Soft Ltd., Kharkiv, Ukraine).

### 2.3. Ecological Niche Modelling (ENM)

Many methods have been used for ENM, but the maximum entropy algorithm implemented in the Maxent software is suggested to perform better than other algorithms [34,35,36,37]. In our study, we used the machine learning algorithm of the Maxent tool [38]. It estimates suitable and unsuitable areas for species based on maximum entropy for presence-only and environmental data [34] and is less sensitive to sample size [39]. Small samples are a significant limitation for building an ecological niche model. We use the recommendations to optimise the model for small samples [40,41,42,43] in order to obtain the most statistically significant and helpful results based on a few sites.

#### 2.3.1. Occurrence Data

**Figure 2:**
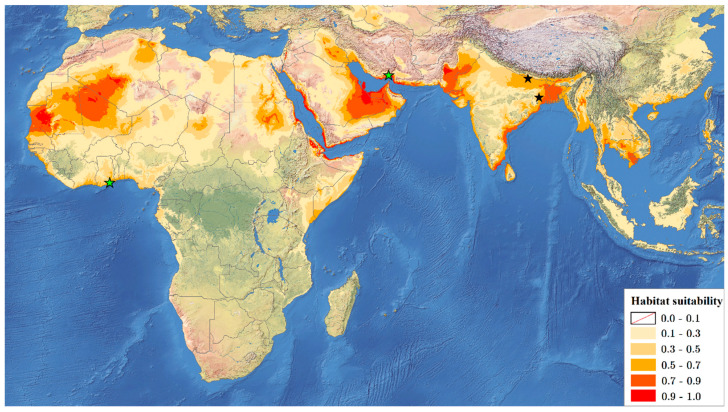
Maxent ecological niche modelling results for *Recaredus rex*. Red and dark orange colours mark areas with high habitat suitability for the species. Literature sites (India) are marked with black stars, while new locations (Iran, Ghana) are marked with green stars.

To model the niche in Maxent, all available information on the presence of *R. rex* (two sites from India [7] and two new sites described in this paper—one from Ghana and one from Iran; Figure 2) were used. The distance between the sites in India is over 480 km; therefore, there is no risk of spatial autocorrelation. The data were georeferenced in Google Earth 9.158.0.0 [44].

#### 2.3.2. Environmental Variables

WorldClim 2.1 climate data for 1970–2000 [45] were used as environmental variables: 19 bioclimatic variables, elevation data, monthly climate data for precipitation, solar radiation, and minimum, maximum, and mean temperature. Both versions of the variables (with WGS84 Coordinate Reference System), 30 arc-seconds spatial resolution and 2.5 arc-minutes spatial resolution, were converted to ASCII format in the ArcGIS Desktop 10.7.1 software [46].

Variable selection was performed to exclude variables that were highly correlated. First, in ArcGIS, the variables with 2.5 arc-minutes spatial resolution were normalised to compare variables with different units. Then, the MaxentVariableSelection vignette [47] for R 4.0.0 [48] was used to extract environmental variables for occurrence and background locations (background data were generated by Maxent 3.4.1 software), removing highly correlated variables (contribution threshold 5, correlation threshold 0.8) and selection of most accurate beta-multiplier settings. Thirty arc-seconds version of selected variables (Table S1) was used in Maxent analysis.

#### 2.3.3. Model Optimisation and Maxent Settings

Maxent software was used to predict *R. rex* presence probability (main settings: auto features, 25 random test percentages, ten bootstrap replicates, maximum test sensitivity plus specificity threshold rule). Modelling was conducted for beta-multiplier from 1.0 to 3.5 (in increments of 0.5). Even though the MaxentVariableSelection vignette indicated 1.0 as the most accurate beta-multiplier, the beta-multiplier 1.5 was selected as it gave a lower standard deviation for the receiver operating characteristic curve and environmental variables response curves, with the same average training AUC (average area under the curve) for the replicate runs. Higher beta-multipliers generated overfitted models.

### 2.4. Output Visualisation and ENM Map Preparation

The point-wise mean picture of the model, obtained as a result of calculations in Maxent, was converted in ArcGIS to TIFF format. The results and occurrence data were plotted on the Natural Earth map [49] with the world administrative boundaries [50]. The jackknife test plot was prepared in R. The environmental variables response curves were generated in Maxent.

### 2.5. Tribal Classification of the Subfamily Tinginae

Since there is no shared view on the tribal classification of the subfamily Tinginae, several different approaches to this problem exist (a detailed review can be found in [13]). Sometimes, the subfamily is not subdivided into lower taxonomic units, e.g., [51,52], or only two tribes, Phatnomini and Tingini (including genera of Litadeini and Ypsotingini) [53,54,55] are recognised. Some authors, however, accept its traditional subdivision into three tribes, namely Ypsotingini, Tingini, and Litadeini [7,11,12,33]. Nevertheless, the complete list of all previously suggested intra-subfamilial taxa of the Tinginae is much longer; they are summarised in the World [11] and the Palaearctic [52] family catalogues.

For the present study, we accept the conventional grouping of the Tinginae genera into three tribes (Ypsotingini, Tingini, Litadeini), with the Acalyptaini, recently recognised as a separate tribe for the genera excluded from the Tingini [13].

## 3. Results and Discussion

### 3.1. Material Studied, First Country Records and Geographic Coordinates

**INDIA**: West Bengal, Pareshnath, 4.000–4.400 ft, 12.iv.1909 (Zoological Survey of India, Kolkata; not examined, see [7]).

**INDIA**: 1 ex.: 14. VIII. 1910, Potato tuber, Mullyah, Bettiah, J. R. No: 927, l. no: 853; Recaredus, A. Hakk det., 1. 1. 63; 1 ex.: 17. VIII. 1910, Potato tuber, Mullyah, Bettiah, J. R. No: 927, l. no: 853; Recaredus, A. Hakk det., 1. 1. 63; 1 ex.: Bettiah, Behar and Orissa, On stored potatoes, H. L. Dutt, Recaredus, A. Hakk det., 1. 1. 63, coll. unknown (NPCI).

**GHANA**: 1 ex.: Legon, Botanical Garden, 22–28 IV 1965, at light, leg. M. J. Prószyński, 1963 (ZMPA). **First country record; new to tropical Africa.**

**IRAN**: 2 exx.: S. Iran, Abbassi, Bang-e Tang, 6 km W. of Geno, 410 m, 50 km N. of Bandar Abbas, 7–9. 05. 1977, Loc. no 323, Exp. Nat. Mus. Praha—Figure 1 (NMPC). **First country record; new to the Palaearctic Region.**

We provide all the geographic coordinates for each locality used for ENM in Table 1.

### 3.2. Ecological Niche Modelling

Maxent’s modelling results (Figure 2) indicate very high habitat suitability for *R. rex* along the Middle Eastern and Northeast African coastlines, bordering the Red Sea, Arabian Sea, and the Persian Gulf. Good environmental conditions for this species can be noted primarily along the eastern shore of the Indian Peninsula, in Bangladesh, in the Indus Valley Plain of Pakistan, in Saudi Arabia’s Al-Ahsa Governorate, and in the western part of the Sahara Desert ecoregion (in Mauritania, Mali and Algeria).

**Figure 3:**
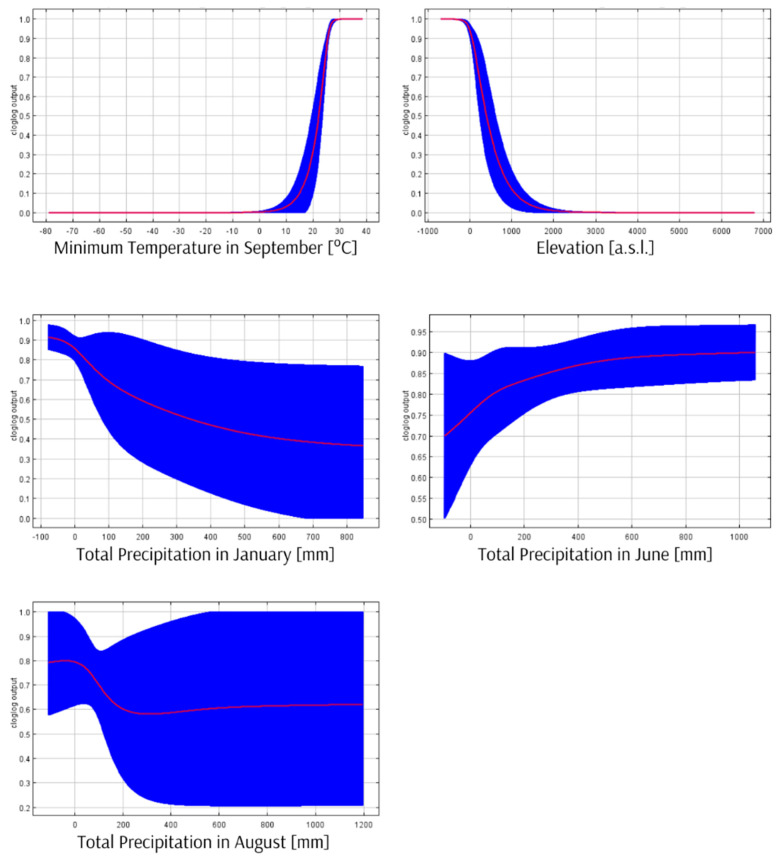
The environmental variables response curves. The mean response of the 10 replicate Maxent runs (red) and the mean +/− one standard deviation (blue) show how each environmental variable affects the Maxent prediction.

**Figure 4:**
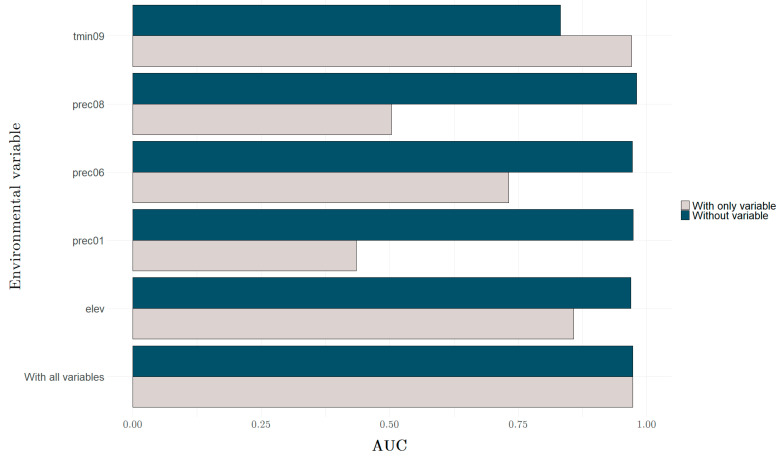
The jackknife test of variable importance. The grey colour indicates using a variable in isolation, and the blue colour specifies the modelling without the selected variable. The higher the score for the first (with only variable) and the lower for the second (without variable), the more critical the selected environmental variable is for the model (AUC—average area under the curve).

The mean AUC for ten replicate Maxent runs is 0.990, and the standard deviation is 0.003. The environmental variables response curves (Figure 3) and the jackknife test of variable importance (Figure 4) indicate that the most substantial impact on the Maxent prediction has a minimum temperature in September—a decrease in the average temperature this month below 27 °C significantly reduces the habitat suitability for this species. The elevation is also essential—the suitability of a location is better in the case of areas located at or below sea level.

The obtained results indicate that the most crucial factor contributing to the high level of habitat suitability for *R. rex* is the temperature in September above 27 °C, especially in areas with low altitudes. The areas with the best environmental conditions for *R. rex* indicated by Maxent can be a starting point for further searches for other specimens of this species.

### 3.3. Systematic Position and New Tribal Assignment

When Golub and Golub [13] restored the tribe Acalyptaini, they provided a set of diagnostic morphological characters enabling its separation from other tribes of the subfamily Tinginae. All of these were also found in *R*. *rex*, as follows: the head with two frontal tubercles (the median spine or tubercle is lacking); the buccal laminae not closed anteriorly; the paranota only slightly oblique; the posterior process of the pronotum flat, and the opening of the metathoracic scent glands without peritreme.

Moreover, the structure of *aedeagus* in *R*. *rex*, especially its bifurcate *ductus seminis* and small *endosomal diverticula* ([7], and Figure 1D–F), suggest a close relation of *Recaredus* to the four genera constituting at present the tribe Acalyptaini (*Acalypta*, *Derephysia*, *Dictyonota*, and *Kalama*) [13]. Though their male genitalia were not analysed when the tribe was restored [13], we consider bifurcate *ductus seminis* and small *endosomal diverticula* as good diagnostic characteristics for this tribe [7,33].

Therefore, considering all the characters mentioned above, we propose transferring the genus *Recaredus* from the tribe Ypsotingini to the Acalyptaini (**new tribal assignment**).

### 3.4. Taxonomy

Order **Hemiptera** Linnaeus, 1758

Suborder **Heteroptera** Latreille, 1810 

Infraorder **Cimicomorpha** Leston, Pendergrast et Southwood, 1954 

Superfamily **Tingoidea** Laporte, 1832 

Family **Tingidae** Laporte, 1832 

Subfamily **Tinginae** Laporte, 1832 

Tribe **Acalyptaini** Blatchley, 1926

Acalyptini Blatchley, 1926: 451, 479. Junior homonym of the Acalyptini Thomson, 1859 (Coleoptera, Curculionidae) [14]. Type genus: *Acalypta* Westwood, 1840.

**Included genera: *Acalypta*** Westwood, 1840. Type species: *Tingis carinata* Panzer, 1806. ***Derephysia*** Spinola, 1837. Type species: *Tingis foliacea* Fallén, 1807. ***Dictyonota*** Curtis, 1827. Type species: *Dictyonota strichnocera* Fieber, 1844. ***Kalama*** Puton, 1876. Type species: *Campylostira* (*Kalama*) *coquereli* Puton, 1876. ***Recaredus*** Distant, 1909 (**new tribal assignment**). Type species: *Recaredus rex* Distant, 1909.

Urn:lsid:zoobank.org:pub: 5FA4989B-2458-41BF-B4B2-519A531AB15B

### 3.5. Key to the World Genera of the Tribe Acalyptaini 

A key to all genera classified currently within the tribe Acalyptaini is provided below. It was adapted from [7,13] and modified adequately to the results of the present study.

**1.** Each hemelytron with tectiform or almost vesicular elevation. Areolae of hemelytra large ([13], p. 232, Figures 10–18) (Figure 1A–B) … **2**

- Hemelytra flat, without tectiform or vesicular elevation [13]. Areolae of hemelytra of moderate size ([13], p. 231, Figures 1–3) … **3**

**2.** Subcostal area with a single row of cells ([13], p. 232, Figures 10–12) … ***Derephysia*** Spinola, 1837

- Subcostal area multiseriate, with three rows of cells ([7], p. 598, Figure 1) ... ***Recaredus*** Distant, 1909

**3.** Head with two frontal spines only [13]. Antennae thin, without tubercles bearing seta on their apices [13]; antennal segment IV distinctly thicker than segment III ([13], p. 321, Figures 1, 4 and 7) … ***Acalypta*** Westwood, 1840

- Head, besides two frontal spines, usually with two additional occipital spines or tubercles visible from above or covered dorsally by areolate hood (vesicula); but if occipital spines absent (in some *Kalama* species), then antennae thick, with distinct and often large tubercles bearing seta apically; antennal segment IV not thicker or insignificantly thicker than segment III [13] … **4**

**4.** Occipital spines always present, noticeably elongated and usually protruding beyond posterior margin of eyes [13]. Preorbital part of the head often noticeably elongated ([13], p. 231, Figures 2, 5 and 8) … ***Dictyonota*** Curtis, 1827

- Occipital spines absent or very small, tuberculate, not elongate, located far behind the posterior margin of eyes [13]. Preorbital part of head short ([13], p. 231, Figures 3, 6 and 9) … ***Kalama*** Puton, 1876.

### 3.6. Distribution and Biology

To date, *R*. *rex* is known only from two localities in India [1,7] and has always been considered an Oriental taxon [1,5,7,8,9,10,11]. Two new records, one from the Palaearctic region and one from tropical Africa, along with the ENM results, suggest a more widespread distribution of this lace bug. The very high habitat suitability for *R. rex* in some areas of Africa, the Near East, and Southeast Asia (Figure 2) indicates its possible Palaeotropical distribution.

Nevertheless, almost nothing is known about the species’ biology and feeding habits. Nonetheless, the collected data (“potato tubers” and “on stored potatoes” [7]) might indicate that this species is somehow closely related to Solanaceae. This is a little surprising since *Solanum tuberosum* Linnaeus, 1753, as well as all other species of Solanaceae, are reported as host plants only for New World lace bugs [11]. Therefore, when the predicted Palaeotropical distribution of *R*. *rex* is considered, it seems reasonable to believe it is an oligo- or polyphagous species.

Furthermore, even though lace bugs are predominantly mono- or oligophagous [11,51,55], several species can feed on plants of various taxonomic groups. Significantly, it is worth mentioning the Ivy lace bug, *Derephysia foliacea* (Fallén, 1807), a polyphagous species [11,52], which is classified within the tribe Acalyptaini [13], the same as *R*. *rex* is in this paper. However, one can also find a widely polyphagous lace bug, e.g., *Cochlochila bullita* (Stål, 1873), that has a Palaeotropical distribution [11,52,56,57,58,59,60,61], the same as suggested for *R*. *rex* in this paper. The predicted areas of *R*. *rex* distribution and indicated possible feeding habits could be a starting point for future studies on this species.

## 4. Conclusions

1. The lace bug genus *Recaredus*, based on the diagnostic characters provided for the tribe Acalyptaini and the structure of *aedeagus,* is transferred from the tribe Ypsotingini to the Acalyptaini.
2. *Recaredus rex*, a lace bug species only known from localities distributed within the Oriental region, are recorded for the first time in the Palaearctic region and the Afrotropics.
3. Ecological niche modelling results and new records of species suggest its possible Palaeotropical distribution.
4. The areas with the best environmental conditions for *R. rex* indicated by Maxent can be a starting point for further searches for specimens of this species. It might help verify the hypothesis of its Palaeotropical distribution and its oligo- or polyphagy.

## Figures and Tables

**Table 1:** Geographic coordinates for each locality where *R*. *rex* was collected.

Position Number on the Map	Country	Locality Data	Coordinates
1	India	West Bengal: Pareshnath, Bankura district	22°57′21.3″ N, 86°44′51.4″ E
2	India	Bihar State: Bettiah, West Champaran district	26°48′05″ N, 84°30′10″ E
3	Iran	Hormozgan province: Abbassi, Bang-e Tang	27°27′ N, 56°18′ E
4	Ghana	Accra Metropolis district: Legon	5°39′ N, 0°11′ W

## Supplementary Materials

The following supporting information can be downloaded at: https://www.mdpi.com/article/10.3390/insects13060558/s1, Table S1. Environmental variables for occurrence and background locations used in Maxent analysis.

## References

[1] Distant W.L. (1909). Rhynchota (Heteroptera) from British India. Ann. de La Sociètè Entomol. de Belg..

[2] Saint Isidore (of Seville) (1970). Isidore of Seville, Historia de regibus Gothorum, Vandalorum et Suevorum, chapter 54. Isidore of Seville’s History of the Goths, Vandals, and Suevi.

[3] Christys A. (2002). Christians in Al-Andalus, 711-1000.

[4] Wolf K.B. (1990). John of Biclaro, Chronicle, 91. Conquerors and Chroniclers of Early Medieval Spain.

[5] Distant W.L. (1910). Rhynchota–Vol. V. Heteroptera: Appendix. The fauna of British India, including Ceylon and Burma.

[6] Imaginary Portrait of Reccared I by Dióscoro Puebla. Oil on Canvas (1857). Museo del Prado, Madrid, Spain. Public Domain. https://commons.wikimedia.org/w/index.php?curid=48073472.

[7] Lis B., Parveen S., Ramamurthy V.V. (2013). Redescription of the Oriental lace-bug *Recaredus rex* Distant, 1909 (Hemiptera: Tingidae: Tinginae), and its new tribal assignment, with a key to Ypsotingini. Zootaxa.

[8] Monte O. (1947). Gêneros e genótipos dos tingídeos do mundo. Papéis Avulsos De Zool..

[9] Drake C.J. (1950). Concerning the Cantacaderinae of the World (Hemiptera: Tingidae). Arthropoda.

[10] Drake C.J., Ruhoff F.A. (1960). Lace-bug genera of the World (Hemiptera: Tingidae). Proc. United States Natl. Mus..

[11] Drake C.J., Ruhoff F.A. (1965). Lacebugs of the World: A Catalog (Hemiptera: Tingidae). USA Natl. Mus. Bull..

[12] Froeschner R.C. (2001). Lace Bug Genera of the World, II: Subfamily Tinginae: Tribes Litadeini and Ypsotingini (Heteroptera; Tingidae). Smithson. Contrib. Zool..

[13] Golub V.B., Golub N.V. (2019). On the status of the genera complex *Acalypta*, *Dictyonota*, *Kalama* and *Derephysia* (Heteroptera: Tingidae: Tinginae) having common morphological and karyological features. Zoosystematica Ross..

[14] Gapon D.A., Golub V.B., Knudson A.H. (2019). Case 3813—Acalyptini Thomson, 1859 (Hexapoda, Coleoptera) and Acalyptini Blatchley, 1926 (Hexapoda, Heteroptera): Proposed removal of homonymy by emendation of the latter name to Acalyptaini. Bull. Zool. Nomencl..

[15] Peterson A.T., Soberón J., Pearson R.G., Anderson R.P., Martínez-Meyer E., Nakamura M., Araújo M.B. (2011). Ecological Niches and Geographic Distributions (MPB-49) 2011.

[16] Warren D.L., Seifert S.N. (2011). Ecological niche modeling in Maxent: The importance of model complexity and the performance of model selection criteria. Ecol. Appl..

[17] Zielińska A., Lis J.A. (2020). Can *Nysius huttoni* F.B. White, 1878 (Hemiptera: Heteroptera: Lygaeidae), a species alien to Europe, have in Poland conditions conducive to existence?. Heteroptera Pol.-Acta Faun..

[18] Zielińska A., Lis B. (2020). Evaluation of the possibilities of potential expansion of the oak lace bug Corythucha arcuata (Say, 1832), an invasive species of Tingidae (Hemiptera: Heteroptera), into the territory of Poland. Heteroptera Pol.-Acta Faun..

[19] Olivera L., Minghetti E., Montemayor S.I. (2021). Ecological niche modeling (ENM) of *Leptoglossus clypealis* a new potential global invader: Following in the footsteps of *Leptoglossus occidentalis*?. Bull. Entomol. Res..

[20] Streito J.-C., Chartois M., Pierre É., Dusoulier F., Armand J.-M., Gaudin J., Rossi J.-P. (2021). Citizen science and niche modeling to track and forecast the expansion of the brown marmorated stinkbug *Halyomorpha halys* (Stål, 1855). Sci. Rep..

[21] Zhu G.-P., Ye Z., Du J., Zhang D.-L., Zhen Y.-h., Zheng C.-g., Zhao L., Li M., Bu W.-J. (2016). Range wide molecular data and niche modeling revealed the Pleistocene history of a global invader (*Halyomorpha halys*). Sci. Rep..

[22] Solhjouy-Fard S., Sarafrazi A., Moeini M.M., Ahadiyat A. (2013). Predicting habitat distribution of five heteropteran pest species in Iran. J. Insect Sci..

[23] Montemayor S.I., Dellapé P.M., Melo M.C. (2015). Predicting the potential invasion suitability of regions to cassava lacebug pests (Heteroptera: Tingidae: Vatiga spp.). Bull. Entomol. Res..

[24] Zhu G.-p., Liu G.-q., Bu W.-J., Lis J.A. (2013). Geographic distribution and niche divergence of two stinkbugs, *Parastrachia japonensis* and *Parastrachia nagaensis*. J. Insect Sci..

[25] Chłond D., Bugaj-Nawrocka A. (2015). Distribution Pattern and Climate Preferences of the Representatives of the Cosmopolitan Genus *Sirthenea* Spinola, 1840 (Heteroptera: Reduviidae: Peiratinae). PLoS ONE.

[26] Parra-Henao G., Suárez-Escudero L.C., González-Caro S. (2016). Potential Distribution of Chagas Disease Vectors (Hemiptera, Reduviidae, Triatominae) in Colombia, Based on Ecological Niche Modeling. J. Trop. Med..

[27] Ye Z., Chen D.-y., Yuan J.-J., Zheng C.-g., Yang X., Wang W.-w., Zhang Y.-y., Wang S., Jiang K., Bu W.-J. (2020). Are population isolations and declines a threat to island endemic water striders? A lesson from demographic and niche modelling of *Metrocoris esakii* (Hemiptera: Gerridae). Mol. Ecol..

[28] Bugaj-Nawrocka A., Sawka-Gądek N., Chłond D. (2020). Prediction of hybridisation zones of selected species of the genus *Platymeris* (Hemiptera: Reduviidae) supported by laboratory crossbreeding. Austral Entomol..

[29] Minghetti E., Olivera L., Montemayor S.I. (2020). Ecological niche modelling of *Gargaphia decoris* (Heteroptera), a biological control agent of the invasive tree *Solanum mauritianum* (Solanales: Solanaceae). Pest Manag. Sci..

[30] Lis J.A., Zielińska A., Lis B. (2022). Ecological niche modelling and first records from Namibia and Zimbabwe validate the amphi-equatorial distribution of *Byrsinus pseudosyriacus* (Hemiptera: Heteroptera: Cydnidae). Afr. J. Ecol..

[31] Fan S., Chen C., Zhao Q., Wei J., Zhang H. (2020). Identifying Potentially Climatic Suitability Areas for *Arma custos* (Hemiptera: Pentatomidae) in China under Climate Change. Insects.

[32] Hoberlandt L. (1983). Results of the Czechoslovak-Iranian entomological expeditions to Iran. Introduction to the Third expedition 1977. Acta Entomol. Musei Natl. Pragae.

[33] Lis B. (2004). Comparative studies on the ductus seminist of aedeagus in Tingoidea (Hemiptera: Heteroptera). Pol. J. Entomol..

[34] Phillips S.J., Anderson R.P., Schapire R.E. (2006). Maximum entropy modeling of species geographic distributions. Ecol. Model..

[35] Elith J., Graham C.H., Anderson R.P., Dudík M., Ferrier S., Guisan A., Hijmans R.J., Huettmann F., Leathwick J.R., Lehmann A. (2006). Novel methods improve prediction of species’ distributions from occurrence data. Ecography.

[36] Elith J., Phillips S.J., Hastie T., Dudík M., Chee Y.E., Yates C.J. (2011). A statistical explanation of MaxEnt for ecologists. Divers. Distrib..

[37] Ortega-Huerta M.A., Peterson A.T. (2008). Modeling ecological niches and predicting geographic distributions: A test of six presence-only methods. Rev. Mex. Biodivers..

[38] Phillips S.J., Dudík M., Schapire R.E. (2022). Maxent Software for Modeling Species Niches and Distributions (Version 3.4.1).

[39] Wisz M.S., Hijmans R.J., Li J., Townsend Peterson A., Graham C.H., Guisan A., NCEAS Predicting Species Distributions Working Group (2008). Effects of sample size on the performance of species distribution models. Divers. Distrib..

[40] Bean W.T., Stafford R., Brashares J.S. (2012). The effects of small sample size and sample bias on threshold selection and accuracy assessment of species distribution models. Ecography.

[41] Shcheglovitova M., Anderson R.P. (2013). Estimating optimal complexity for ecological niche models: A jackknife approach for species with small sample sizes. Ecol. Model..

[42] Li Y., Ding C. (2016). Effects of sample size, sample accuracy and environmental variables on predictive performance of MaxEnt model. Pol. J. Ecol..

[43] Pearson R.G., Raxworthy C.J., Nakamura M., Townsend Peterson A. (2007). Predicting species distributions from small numbers of occurrence records: A test case using cryptic geckos in Madagascar. J. Biogeogr..

[44] Google Earth 9.158.0.0. http://Earth.Google.Com/Web/.

[45] Fick S.E., Hijmans R.J. (2017). WorldClim 2: New 1km spatial resolution climate surfaces for global land areas. Int. J. Climatol..

[46] ESRI (2022). ArcGIS Desktop: Realease 10.7.1.

[47] Jueterbock A., Smolina I., Coyer J.A., Hoarau G. (2016). The fate of the Arctic seaweed *Fucus distichus* under climate change: An ecological niche modeling approach. Ecol. Evol..

[48] R Core Team (2022). R: A Language and Environment for Statistical Computing.

[49] Natural Earth Natural Earth I with Shaded Relief, Water, and Drainages. https://www.naturalearthdata.com/downloads/10m-raster-data/10m-natural-earth-1/.

[50] Open Data Soft World Administrative Boundaries (by World Food Programme (UN Agency)). https://public.opendatasoft.com/explore/dataset/world-administrative-boundaries/information/.

[51] Péricart J. (1983). Hémiptères Tingidae euro-mediterranéens. Faune de France 69.

[52] Péricart J., Golub V.B. (1996). Superfamily Tingoidea Laporte, 1832. Catalogue of the Heteroptera of the Palaearctic Region. Cimicomorpha I.

[53] Guilbert E. (2004). Do larvae evolve the same way as adults in Tingidae (Insecta: Heteroptera)?. Cladistics.

[54] Guilbert E., Damgaard J., D’Haese C.A. (2014). Phylogeny of the lacebugs (Insecta: Heteroptera: Tingidae) using morphological and molecular data. Syst. Entomol..

[55] Schuh R.T., Weirauch C.H. (2020). True bugs of the World (Hemiptera: Heteroptera). Classification and Natural History.

[56] Wert Palaniswami M.S., Pillai K.S. (1983). Biology of *Cochlochila bullita* a pest on Chinese potato. J. Root Crops.

[57] Stonedahl G., Dolling W., DuHeaume G. (1992). Identification guide to common tingid pests of the World (Heteroptera: Tingidae). Int. J. Pest Manag..

[58] Göllner-Scheiding U. (2004). Die Tingidae (Netzwanzen) der Äthiopis (Insecta, Heteroptera: Tingoidea). Katalog der afrikanischen Arten. Nova Supplemcnta Entomol. Keltern.

[59] Deckert J., Göllner-Scheiding U. (2006). Lace bugs of Namibia (Heteroptera, Tingoidea, Tingidae). Denisia.

[60] Schaefer C.W., Panizzi A.R. (2010). Heteroptera of Economic Importance.

[61] Smith-Pardo A.H. (2013). The lace bug *Cochlochila bullita* (Stål) (Heteroptera: Tingidae), an important pest of cultivated herbs in Asia, intercepted at U.S. ports of entry. Bol. Del Mus. Entomológico Fr. Luís Gallego.

