# Supplementary material for: The King’s Lace Bug Recaredus rex Distant, 1909 (Hemiptera: Heteroptera: Tingidae): Systematic Position, First Palaearctic and Afrotropical Records, and Ecological Niche Modelling
Source: Insects. 2022 Jun 19;13(6):558. doi: 10.3390/insects13060558 (PMC9225140; doi:10.3390/insects13060558)
Supplement: Supplementary file 1 [file insects-13-00558-s001.zip › insects-1753256-SI.pdf]

Table S1

VariableSelectionMaxAUCTest

Test Model	Contributions 1	Correlation 1	Contributions 2	Correlation 2	Contributions 3	Correlation 3
bio10n	0	NA	NA	NA	NA	NA
bio11n	0	NA	NA	NA	NA	NA
bio12n	0	NA	NA	NA	NA	NA
bio13n	0	NA	NA	NA	NA	NA
bio14n	0.3061	NA	NA	NA	NA	NA
bio15n	0	NA	NA	NA	NA	NA
bio16n	0,0003	NA	NA	NA	NA	NA
bio17n	2.5050	NA	NA	NA	NA	NA
bio18n	0.0102	NA	NA	NA	NA	NA
bio19n	2.5748	NA	NA	NA	NA	NA
bio1n	0	NA	NA	NA	NA	NA
bio2n	0	NA	NA	NA	NA	NA
bio3n	0	NA	NA	NA	NA	NA
bio4n	0	NA	NA	NA	NA	NA
bio5n	0	NA	NA	NA	NA	NA
bio6n	0	NA	NA	NA	NA	NA
bio7n	0	NA	NA	NA	NA	NA
bio8n	0	NA	NA	NA	NA	NA
bio9n	0	NA	NA	NA	NA	NA
**elevn**	NA	NA	1.8548	NA	NA	NA
prec10n	0	NA	NA	NA	NA	NA
prec11n	1.4517	NA	NA	NA	NA	NA
prec12n	3.9054	NA	NA	NA	NA	NA
**prec1n**	6.7313000	0.3920597	11.7256000	0.2544369	14.8851000	1.0000000
prec2n	0.7889	NA	NA	NA	NA	NA
prec3n	0.1608	NA	NA	NA	NA	NA
prec4n	0	NA	NA	NA	NA	NA
prec5n	0	NA	NA	NA	NA	NA
**prec6n**	19.4512000	0.4220510	34.4337000	1.0000000	8.6171000	0.2544369
prec7n	0	NA	NA	NA	NA	NA
**prec8n**	17.0887000	0.3438415	0.1007000	NA	NA	NA
prec9n	0	NA	NA	NA	NA	NA
srad10n	0	NA	NA	NA	NA	NA
srad11n	0	NA	NA	NA	NA	NA
srad12n	0	NA	NA	NA	NA	NA
srad1n	0	NA	NA	NA	NA	NA
srad2n	0	NA	NA	NA	NA	NA
srad3n	0	NA	NA	NA	NA	NA
srad4n	10.0633000	0.9268476	NA	NA	NA	NA
srad5n	0.0041	NA	NA	NA	NA	NA
srad6n	0	NA	NA	NA	NA	NA
srad7n	0	NA	NA	NA	NA	NA
srad8n	0	NA	NA	NA	NA	NA
srad9n	0	NA	NA	NA	NA	NA
tavg10n	0	NA	NA	NA	NA	NA
tavg11n	0	NA	NA	NA	NA	NA
tavg12n	0	NA	NA	NA	NA	NA
tavg1n	0	NA	NA	NA	NA	NA
tavg2n	0	NA	NA	NA	NA	NA
tavg3n	0	NA	NA	NA	NA	NA
tavg4n	0	NA	NA	NA	NA	NA
tavg5n	0	NA	NA	NA	NA	NA
tavg6n	0	NA	NA	NA	NA	NA

tavg7n	0	NA	NA	NA	NA	NA
tavg8n	0	NA	NA	NA	NA	NA
tavg9n	0	NA	NA	NA	NA	NA
tmax10n	0	NA	NA	NA	NA	NA
tmax11n	0	NA	NA	NA	NA	NA
tmax12n	0	NA	NA	NA	NA	NA
tmax1n	0	NA	NA	NA	NA	NA
tmax2n	0	NA	NA	NA	NA	NA
tmax3n	0	NA	NA	NA	NA	NA
tmax4n	0	NA	NA	NA	NA	NA
tmax5n	0	NA	NA	NA	NA	NA
tmax6n	0	NA	NA	NA	NA	NA
tmax7n	0	NA	NA	NA	NA	NA
tmax8n	0	NA	NA	NA	NA	NA
tmax9n	0	NA	NA	NA	NA	NA
tmin10n	0.0012	NA	NA	NA	NA	NA
tmin11n	0	NA	NA	NA	NA	NA
tmin12n	0	NA	NA	NA	NA	NA
tmin1n	0	NA	NA	NA	NA	NA
tmin2n	0	NA	NA	NA	NA	NA
tmin3n	0	NA	NA	NA	NA	NA
tmin4n	4.6893000	NA	NA	NA	NA	NA
tmin5n	0.6723	NA	NA	NA	NA	NA
tmin6n	0	NA	NA	NA	NA	NA
tmin7n	0	NA	NA	NA	NA	NA
tmin8n	1.184	NA	NA	NA	NA	NA
**tmin9n**	26.5566000	1.0000000	53.7401000	0.4220510	76.4977000	0.3920597
